# Longitudinal Assessment of High Blood Pressure in Children with Nonalcoholic Fatty Liver Disease

**DOI:** 10.1371/journal.pone.0112569

**Published:** 2014-11-24

**Authors:** Jeffrey B. Schwimmer, Anne Zepeda, Kimberly P. Newton, Stavra A. Xanthakos, Cynthia Behling, Erin K. Hallinan, Michele Donithan, James Tonascia, for the Nonalcoholic Steatohepatitis Clinical Research Network

**Affiliations:** 1 Division of Gastroenterology, Hepatology, and Nutrition, Department of Pediatrics, University of California San Diego, School of Medicine, San Diego, California, United States of America; 2 Department of Gastroenterology, Rady Children's Hospital San Diego, San Diego, California, United States of America; 3 Liver Imaging Group, Department of Radiology, University of California San Diego, School of Medicine, San Diego, California, United States of America; 4 University of California San Diego, Master of Advanced Studies in Clinical Research, San Diego, California, United States of America; 5 Division of Gastroenterology, Hepatology and Nutrition, Cincinnati Children's Hospital Medical Center, Cincinnati, Ohio, United States of America; 6 Department of Pathology, Sharp Medical Center, San Diego, California, United States of America; 7 Johns Hopkins Bloomberg School of Public Health, Baltimore, Maryland, United States of America; Institute of Medical Research A Lanari-IDIM, University of Buenos Aires-National Council of Scientific and Technological Research (CONICET), Argentina

## Abstract

**Objective:**

Nonalcoholic fatty liver disease (NAFLD) affects 9.6% of children and may put these children at elevated risk of high blood pressure and subsequent cardiovascular morbidity and mortality. Therefore, we sought to determine the prevalence of and risk factors for high blood pressure in children with NAFLD.

**Methods:**

Cohort study performed by the NIDDK NASH Clinical Research Network. There were 484 children with NAFLD ages 2 to 17 at enrollment; 382 children were assessed both at enrollment and 48 weeks afterwards. The main outcomes were high blood pressure at baseline and persistent high blood pressure at both baseline and 48 weeks.

**Results:**

Prevalence of high blood pressure at baseline was 35.8% and prevalence of persistent high blood pressure was 21.4%. Children with high blood pressure were significantly more likely to have worse steatosis than children without high blood pressure (mild 19.8% vs. 34.2%, moderate 35.0% vs. 30.7%, severe 45.2% vs. 35.1%; *P* = 0.003). Higher body mass index, low-density lipoprotein, and uric acid were independent risk factors for high blood pressure (Odds Ratios: 1.10 per kg/m^2^, 1.09 per 10 mg/dL, 1.25 per mg/dL, respectively). Compared to boys, girls with NAFLD were significantly more likely to have persistent high blood pressure (28.4% vs.18.9%; *P* = 0.05).

**Conclusions:**

In conclusion, NAFLD is a common clinical problem that places children at substantial risk for high blood pressure, which may often go undiagnosed. Thus blood pressure evaluation, control, and monitoring should be an integral component of the clinical management of children with NAFLD.

## Introduction

High blood pressure and nonalcoholic fatty liver disease (NAFLD) are two emerging clinical problems in children closely related to the epidemic of childhood obesity. NAFLD is now the most common cause of chronic liver disease in children in the United States with an estimated prevalence of 9.6%. [1] The prevalence of high blood pressure is estimated to be between 2 and 5% among children in the United States. [2], [3] High blood pressure in childhood is likely to persist into adulthood [4], [5] and is a risk factor in adulthood for atherosclerosis and coronary heart disease. [6]

NAFLD itself has been linked to cardiovascular disease in both children and adults. [7]–[9] In adults with NAFLD, cardiovascular disease is the leading cause of death. [10], [11] In children with NAFLD, studies have reported high blood pressure as part of the metabolic syndrome; however, blood pressure has not been evaluated as the focal point of any of these studies. [9], [12], [13] Thus, many questions remain about the prevalence of high blood pressure and its associated risk factors in children with NAFLD. Moreover, there have been no longitudinal studies of blood pressure among children with NAFLD. Therefore we performed a multi-center, longitudinal cohort study with the following study aims: 1) to determine the prevalence of high blood pressure in children with NAFLD in relation to demographic and key clinical risk factors, and 2) to determine the rate and risk factors of persistent (over 48 weeks) high blood pressure in children with NAFLD.

## Methods

### Study population

The National Institute of Diabetes and Digestive and Kidney Diseases (NIDDK) NASH Clinical Research Network (NASH CRN) enrolled children with NAFLD in longitudinal cohort studies (Database and Database 2; NCT01061684) and a randomized controlled trial (TONIC; NCT00063635). These studies have been described [14], [15] and were performed at 13 pediatric clinical centers across the United States (see appendix for list). NAFLD Database began enrollment in September 2004, TONIC in August 2005 and Database 2 in October 2009. Children completed annual visits with comprehensive anthropometric and laboratory measurements described below. For this analysis, we included children who were <18 years of age at baseline with NAFLD. A diagnosis of NAFLD was based on liver histology with ≥5% of hepatocytes containing macrovesicular fat and exclusion of other causes of chronic liver disease by clinical history, laboratory studies, and histology. [16] For the baseline analysis, we excluded children with underlying renal disease, those without a histologic diagnosis of NAFLD, or missing blood pressure at baseline. For the longitudinal analysis, we excluded those without follow-up data at 48 weeks.

### Ethics Statement

All studies were approved by the Institutional Review Boards at each participating center. Written informed consent was obtained from parents/guardians and written assent was obtained from children.

### Liver Histology

All liver biopsy specimens were stained with hematoxylin-eosin and Masson's trichrome stains, and reviewed and scored centrally by the Pathology Committee according to the published NASH Clinical Research Network scoring system. [17] The Pathology Committee was blinded to any clinical or demographic information. Steatosis was graded according to the percentage of hepatocytes that contained fat droplets as follows: grade 0, none: <5%; grade 1, mild: 5 to 33%; grade 2, moderate: 34 to 66%; and grade 3, severe:> 66%. Fibrosis was staged as follows: a) stage 1a – mild zone 3 perisinusoidal fibrosis requiring trichrome stain; b) stage 1b – moderate zone 3 perisinusoidal fibrosis not requiring trichrome stain; c) stage 1c – portal/periportal fibrosis only; c) stage 2 –zone 3 perisinusoidal fibrosis and periportal fibrosis; d) stage 3 – bridging fibrosis; and e) stage 4 – cirrhosis. Liver biopsies were diagnosed as steatohepatitis, borderline steatohepatitis or NAFLD but not steatohepatitis, based on the aggregate presence and degree of the individual histologic features of fatty liver disease. Although no single histologic feature is considered diagnostic of NASH, a typical set of minimum criteria would include macrovesicular steatosis (more than 5%), lobular inflammation and hepatocyte injury as manifest by ballooning degeneration. Borderline cases demonstrated a lesser degree of one or more findings. Cases determined to be NAFLD but not steatohepatitis NASH show steatosis with no or minimal lobular inflammation. The assignment of a diagnosis of steatohepatitis, borderline steatohepatitis or NAFLD but not steatohepatitis was made as a consensus agreement of the NASH CRN pathology group at the time of central review of cases.

### Measures

Physical measurements included height, weight, waist circumference, systolic blood pressure, and diastolic blood pressure. Blood pressure was measured and percentiles were computed as instructed in The Fourth Report on the Diagnosis, Evaluation, and Treatment of High Blood Pressure in Children and Adolescents. [18] After 5 minutes of seated rest, blood pressure was measured twice from the right arm of the seated child with an automated sphygmomanometer with 1 minute of rest between measurements. The average of the 2 measures was recorded. Cuff sizes were selected so that the cuff bladder encircled at least 80% of the mid-upper arm per standard protocol. Participants fasted overnight for 12 hours before phlebotomy. Fasting laboratory assays included glucose, insulin, total cholesterol, high-density lipoprotein (HDL) cholesterol, low-density lipoprotein (LDL) cholesterol, triglycerides, alanine aminotransferase (ALT), aspartate aminotransferase (AST), gamma glutamyltransferase (GGT), and uric acid. Body mass index (BMI) was calculated as weight (kg) divided by height (m) squared.

### Case definitions

The definitions for blood pressure in children are based upon the normative distribution of blood pressure in healthy children. [18] High blood pressure is defined as average systolic or diastolic blood pressure that is ≥95^th^ percentile for age, sex, and height. Hypertension is defined as high blood pressure on at least three separate occasions. For this analysis we defined *High blood pressure* as systolic or diastolic blood pressure ≥95th percentile for age, sex and height, or the use of antihypertensive medication for a clinical diagnosis of hypertension. *Persistent high blood pressure* was defined as having high blood pressure at both baseline and at 48 weeks. *Clinical hypertension* was determined by parental or patient report that a child had a clinical diagnosis of hypertension assigned by their treating physician.

### Data Analysis

Descriptive statistics (mean, standard deviation, frequency, and percentages) were used to compare patients with and without elevated blood pressure; P values were determined either from Chi-square tests for categorical variables or from non-parametric two-sample Wilcoxon rank sum tests for continuous variables. Risk factors for high blood pressure were identified using multiple logistic regression models with the presence of high blood pressure as the binary outcome and a candidate set of risk factors: gender, age, race/ethnicity (non-Hispanic white, Hispanic, and non-Hispanic non-white), BMI, GGT, LDL, glucose, insulin, and uric acid. Goodness of fit of the logistic model was assessed using a Hosmer-Lemeshow chi-square test with P>0.05 indicating adequate fit. Parallel analyses were done for risk factors for persistently high blood pressure. Characteristics of those children with and without 48 week follow-up were compared using a logistic regression model of the odds of missing-ness in relation to the risk factors; a Wald test was performed to determine whether set of characteristics differed in those children who were not evaluated at a 48 week follow-up assessment. All analyses assumed nominal, two-sided P values as statistically significant if P≤0.05. Analyses were performed using SAS version 9.3 (SAS Institute) and Stata version 13.1 (StataCorp). Sensitivity analysis of variation in risk factors by gender showed no evidence of effect modification (interaction *P* range from 0.12 to 0.81). Additionally, the set of risk factors was not related to the odds of missing the 48 week follow-up visit (*P* = 0.41).

## Results

### Study population

**Figure 1:**
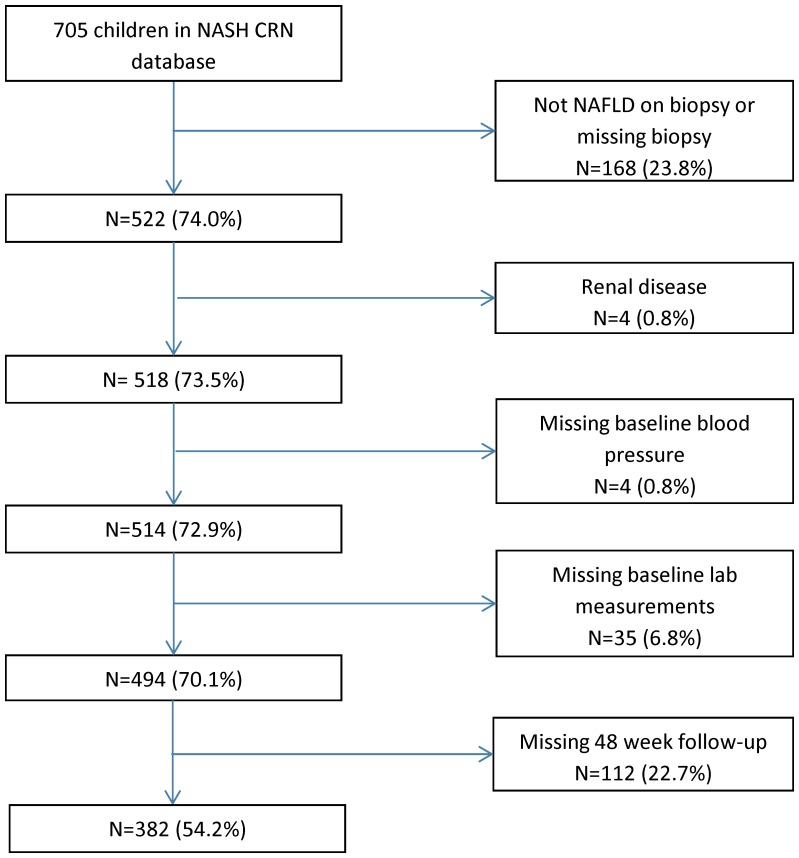
Flowchart shows the application of study inclusion and exclusion criteria.

There were 494 children enrolled in the NASH CRN that met all criteria and were included in the baseline analysis. A study flow chart is shown in **Figure 1**. The demographic and clinical parameters are shown in **Table 1**. The mean age of the participants at baseline was 13.1 years. The mean BMI of participants at baseline was 32.7 kg/m^2^. The distribution of disease severity was: NAFLD but not NASH 27.5% (136/494), borderline NASH 44.7% (221/494) and definite NASH 27.7% (137/494). The majority of participants (358/494) were boys. There was no significant difference between boys and girls with respect to age or race/ethnicity. Boys had a significantly higher BMI Z-score than girls (2.4±0.4 vs 2.2±0.4; *P*<0.001) but no difference in BMI (32.9±6.3 vs 32.6±7.0 kg/m^2^; *P* = 0.33).

**Table 1:** High Blood Pressure in Children with NAFLD—Baseline Characteristics.

	High Blood Pressure^a^			
Characteristics	No	Yes	Total	*P* Value^b^
N (%) or mean ±SD	N = 317	N = 177	N = 494	
**Blood pressure**				
Systolic blood pressure percentile	64.1±25.0	95.8±24.0	75.5±26.0	**<0.0001**
Diastolic blood pressure percentile	53.9±23.6	73.8±21.9	61.0±24.9	**<0.0001**
**Demographics**				
Male	234 (73.8%)	124 (70.1%)	358 (72.5%)	0.37
Age (years)^c^	13.1±2.7	12.9±2.8	13.1±2.7	0.35
<8 years	11 (3.5%)	6 (3.4%)	17 (3.4%)	0.33
8–12 years	141 (44.5%)	91 (51.4%)	232 (47.0%)	
13–17 years	165 (52.1%)	80 (45.2%)	245 (49.6%)	
Race/ethnicity				**0.05**
Non-Hispanic white	85 (26.8%)	63 (35.6%)	148 (30.0%)	
Hispanic	216 (68.4%)	101 (57.1%)	317 (64.2%)	
Other	16 (5.1%)	13 (7.3%)	29 (5.9%)	
**Anthropomorphic**				
BMI z-score	2.2±0.4	2.4±0.4	2.3±0.4	**<0.0001**
BMI (kg/m^2^)	31.6±6.2	34.6±6.5	32.7±6.5	**<0.0001**
**Liver enzymes**				
ALT (U/L)	105.9±84.3	107.1±88.8	106.3±85.8	0.83
AST (U/L)	63.2±48.5	64.6±45.3	63.7±47.3	0.41
GGT (U/L)	42.4±29.9	48.6±34.5	44.6±31.7	**0.004**
**Serum chemistries**				
HDL (mg/dL)	38.5±8.5	38.2±10.1	38.4±9.1	0.17
LDL (mg/dL)	100.2±29.9	106.4±28.7	102.4±29.6	**0.04**
Serum glucose (mg/dL)	87.7±18.1	89.3±15.0	88.3±17.1	**0.05**
Serum insulin (µU/mL)	31.7±38.5	37.4±28.5	33.7±35.3	**0.001**
HOMA-IR^d^	7.0±9.3	8.3±6.7	7.5±8.5	**0.001**
Uric acid (mg/dL)	5.9±1.4	6.3±1.4	6.0±1.4	**0.03**
**Liver Histology**				
Steatosis grade				**0.003**
<33%	108 (34.2%)	35 (19.8%)	143 (29.0%)	
34–66%	97 (30.7%)	62 (35.0%)	159 (32.3%)	
>66%	111 (35.1%)	80 (45.2%)	191 (38.7%)	
Lobular inflammation				0.94
<2 under 20x	173 (54.8%)	95 (53.7%)	268 (54.4%)	
2–4 under 20x	122 (38.6%)	71 (40.1%)	193 (39.2%)	
>4 under 20x	21 (6.7%)	11 (6.2%)	32 (6.5%)	
Ballooning				**0.02**
None	174 (55.1%)	96 (54.2%)	270 (54.8%)	
Few	81 (25.6%)	61 (34.5%)	142 (28.8%)	
Many	61 (19.3%)	20 (11.3%)	81 (16.4%)	
Fibrosis stage				0.47
None	102 (32.4%)	44 (25.0%)	146 (29.7%)	
Zone 3, perisinusoidal or portral/periportal only	121 (38.4%)	73 (41.5%)	194 (39.5%)	
Zone 3, periportal	49 (15.6%)	30 (17.1%)	79 (16.1%)	
Bridging	38 (12.1%)	27 (15.3%)	65 (13.2%)	
Cirrhosis	5 (1.6%)	2 (1.1%)	7 (1.4%)	
Diagnosis				0.42
NAFLD, not NASH	93 (29.3%)	43 (24.3%)	136 (27.5%)	
Borderline NASH	136 (42.9%)	85 (48.0%)	221 (44.7%)	
Definite NASH	88 (27.8%)	49 (27.7%)	137 (27.7%)	

Abbreviations: BMI = body mass index, ALT = alanine aminotransferase, AST =  aspartate aminotransferase, GGT = gamma-glutamyl transpeptidase, HDL = high-density lipoprotein, LDL = low-density lipoprotein, HOMA-IR = homeostasis model of assessment - insulin resistance

a We defined high blood pressure as systolic or diastolic blood pressure ≧95th percentile for age, sex and height or the use of antihypertensive medication. Blood pressure percentiles were computed as instructed in The Fourth Report on the Diagnosis, Evaluation, and Treatment of High Blood Pressure in Children and Adolescents.

b P values determined from chi square tests for categorical variables and from two-sample Wilcoxon rank sum tests for continuous variables due to the presence of non-normality.

c Age range from 2–17 years.

d HOMA-IR units are (mg/dL×IU/mL/405).

### High Blood Pressure at Baseline

The estimated prevalence of high blood pressure at baseline was 35.8% (95% CI 31.7–40.2). As shown in **Table 1**, children with and without high blood pressure did not differ significantly by age, sex or race. Children with high blood pressure had a significantly higher mean BMI than children without high blood pressure (34.6 vs. 31.6 kg/m^2^; *P*<0.0001). Children with high blood pressure also had significantly higher GGT, LDL-cholesterol, serum fasting insulin, and uric acid values. In addition, children with high blood pressure had significantly more severe steatosis (mild: 19.8%, moderate: 35.0%, severe: 45.2%) than children without high blood pressure (mild: 34.2%, moderate: 30.7%, severe: 35.1%, *P* = 0.003). As shown in **Table 2**, each one unit increase in BMI was associated with 10% greater odds of having high blood pressure (95%CI: 6%–14%). There was a significant linear relationship between LDL-cholesterol and odds of high blood pressure (OR [95%CI]: 1.09 per 10 mg/dL [1.02, 1.17]). In addition, for every 1 mg/dL of uric acid there was a 25% increase in the odds of having high blood pressure (95%CI: 6%–48%).

**Table 2:** Clinical, Demographic, and Biochemical Risk Factors for High Blood Pressure at Baseline.

	Odds Ratios (OR) for High Blood Pressure^a^			
	Single variable logistic		Adjusted, Multivariable Logistic	
Risk Factors	OR (95%CI)	*P* Value^b^	OR (95% CI)	*P* Value^b^
**Demographics**				
Male vs Female	0.83 (0.55, 1.25)	0.37	0.71 (0.45, 1.12)	0.14
Age/year	0.98 (0.91, 1.04)	0.47	0.82 (0.75, 0.90)	**<0.001**
Race/ethnicity		**0.05**		0.36
Non-Hispanic white	1.0 (Reference)		1.0 (Reference)	
Hispanic	0.63 (0.42, 0.94)	**0.03**	0.84 (0.53, 1.33)	0.46
Other	1.10 (0.49, 2.44)	0.82	1.51 (0.62, 3.63)	0.36
**Anthropomorphic**				
BMI/(kg/m^2^)	1.08 (1.04, 1.11)	**<0.001**	1.10 (1.06, 1.14)	**<0.001**
**Liver enzymes**				
GGT/(10 U/L)	1.06 (1.00, 1.12)	**0.04**	1.03 (0.96, 1.09)	0.44
**Serum chemistries**				
LDL/(10 mg/dL)	1.07 (1.01, 1.14)	**0.03**	1.10 (1.03, 1.18)	**0.006**
Serum glucose/(10 mg/dL)	1.06 (0.95, 1.18)	0.32	1.07 (0.96, 1.19)	0.25
Serum insulin/(10 µU/mL)	1.05 (0.99, 1.11)	0.11	1.01 (0.96, 1.07)	0.70
Uric acid/(mg/dL)	1.18 (1.03, 1.35)	**0.02**	1.25 (1.05, 1.49)	**0.01**
**Liver histology**				
Steatosis grade>33%	2.11 (1.36, 3.26)	**0.001**	2.26 (1.39, 3.66)	**0.001**
Intercept			0.01 (0.001, 0.08)	**<0.001**
Hosmer-Lemeshow χ^2^ for model fit				0.94

Abbreviations: OR = odds ratio, CI = confidence interval, BMI = body mass index, GGT = gamma-glutamyl transpeptidase, LDL = low-density lipoprotein.

a High blood pressure was defined as systolic or diastolic blood pressure ≧95th percentile for age, sex and height or the use of antihypertensive medication. Blood pressure percentiles were computed as instructed in The Fourth Report on the Diagnosis, Evaluation, and Treatment of High Blood Pressure in Children and Adolescents.

b *P* values and 95% CI were obtained from Wald statistics.

### Persistent High Blood Pressure

The estimated prevalence of persistent high blood pressure was 21.4% (95%CI 17.6–25.9). Girls with NAFLD were significantly more likely to have persistent high blood pressure than boys with NAFLD (28.4% [20.5%–38.0%] vs.18.9% [14.7%–24.0%]). As shown in **Table 3**, children with persistent high blood pressure were more likely to be non-Hispanic white (36.6% vs. 27.7%). Similar to the differences seen at baseline, children with persistent high blood pressure had significantly higher values of GGT, LDL-cholesterol, insulin, and uric acid than children without persistent high blood pressure. There was no significant difference in the severity of any histologic feature between children with and without persistent high blood pressure. In multivariate analysis (**Table 4**), boys with NAFLD had 45% lower odds of having persistent high blood pressure than girls with NAFLD (95%CI: 3–69%). BMI, LDL-cholesterol, and uric acid were all significantly positively associated with the odds of having persistent high blood pressure (OR[95%CI]: 1.10[1.05, 1.15], 1.12[1.03, 1.23], 1.32[1.06, 1.64], respectively). Sensitivity analysis of variation in risk factors by gender showed no evidence of effect modification (interaction P range from 0.12 to 0.81). Additionally, the set of risk factors was not related to the odds of missing the 48 week follow-up visit (P = 0.41).

**Table 3:** Persistently High Blood Pressure in Children with NAFLD—Baseline Characteristics.

	Persistently High Blood Pressure^a^		
Characteristics	No	Yes	*P* Value^b^
N (%) or mean±SD	N = 300	N = 82	
**Blood pressure**			
** **Systolic blood pressure percentile	71.0±25.9	95.6±12.0	**<0.0001**
Diastolic blood pressure percentile	56.6±24.2	73.8±24.7	**<0.0001**
**Demographics**			
Male	227 (75.7%)	53 (64.6%)	**0.05**
Age (years)^c^	13.0±2.7	13.0±2.6	0.85
<8 years	7 (2.3%)	2 (2.5%)	0.96
8–12 years	145 (48.3%)	41 (50.0%)	
13–17 years	148 (49.3%)	39 (47.6%)	
Race/ethnicity			**0.007**
Non-Hispanic white	83 (27.7%)	30 (36.6%)	
Hispanic	205 (68.3%)	43 (52.4%)	
Other	12 (4.0%)	9 (11.0%)	
**Anthropomorphic**			
BMI z-score	2.3±0.5	2.5±0.3	**<0.0001**
BMI (kg/m^2^)	32.1±6.4	35.3±6.1	**<0.0001**
**Liver enzymes**			
ALT (U/L)	106.4±84.9	110.2±82.4	0.77
AST (U/L)	62.8±44.2	69.6±53.1	0.50
GGT (U/L)	43.9±31.8	50.7±36.7	**0.04**
**Serum chemistries**			
HDL (mg/dL)	38.4±8.3	37.8±11.3	0.08
LDL (mg/dL)	100.3±29.6	108.8±29.8	**0.03**
Serum glucose (mg/dL)	88.6±18.2	90.7±17.2	**0.05**
Serum insulin (µU/mL)	33.5±39.3	39.1±32.4	**0.04**
HOMA-IR^d^	7.4±9.5	8.9±7.8	**0.04**
Uric acid (mg/dL)	6.0±1.4	6.4±1.5	**0.03**
**Liver Histology**			
Steatosis grade			0.45
<33%	91 (30.3%)	19 (23.2%)	
34–66%	91 (30.3%)	27 (32.9%)	
>66%	118 (49.3%)	36 (43.9%)	
Lobular inflammation			0.19
<2 under 20x	151 (50.3%)	47 (57.3%)	
2–4 under 20x	133 (44.3%)	28 (34.2%)	
>4 under 20x	16 (5.3%)	7 (8.5%)	
Ballooning			0.44
None	162 (54.0%)	42 (51.2%)	
Few	86 (28.7%)	29 (35.4%)	
Many	52 (17.3%)	11 (13.4%)	
Fibrosis stage			0.58
None	101 (33.8%)	22 (27.2%)	
Zone 3, perisinusoidal or portral/periportal only	114 (38.1%)	30 (37.0%)	
Zone 3, periportal	46 (15.4%)	16 (19.8%)	
Bridging	35 (11.7%)	11 (13.6%)	
Cirrhosis	3 (1.0%)	2 (2.5%)	
Diagnosis			0.83
NAFLD, not NASH	87 (29.0%)	22 (26.8%)	
Borderline NASH	135 (45.0%)	36 (43.9%)	
Definite NASH	78 (26.0%)	24 (29.3%)	

Abbreviations: BMI = body mass index, ALT = alanine aminotransferase, AST =  aspartate aminotransferase, GGT = gamma-glutamyl transpeptidase, HDL = high-density lipoprotein, LDL = low-density lipoprotein, HOMA-IR = homeostasis model of assessment - insulin resistance.

a We defined persistently high blood pressure as systolic or diastolic blood pressure ≧95th percentile for age, sex and height or the use of antihypertensive medication at both baseline and 48 week follow-up. Blood pressure percentiles were computed as instructed in The Fourth Report on the Diagnosis, Evaluation, and Treatment of High Blood Pressure in Children and Adolescents.

b P values determined from chi square tests for categorical variables and from two-sample Wilcoxon rank sum tests for continuous variables due to the presence of non-normality.

c Age range from 2–17 years.

d HOMA-IR units are (mg/dL × IU/mL/405).

**Table 4:** Clinical, Demographic, and Biochemical Risk Factors for Persistently High Blood Pressure.

	Odds Ratios (OR) of Persistently High Blood Pressure^a^			
	Unadjusted		Adjusted, Multivariable Logistic	
Characteristic	OR (95%CI)	*P* Value^b^	OR (95% CI)	*P* Value^b^
**Demographics**				
Male vs female	0.59 (0.35, 0.99)	**0.05**	0.49 (0.27, 0.88)	**0.02**
Age/years	1.00 (0.91, 1.09)	0.98	0.82 (0.73, 0.93)	**0.001**
Race/ethnicity		**0.009**		0.12
Non-Hispanic white	1.0 (Reference)		1.0 (Reference)	
Hispanic	0.58 (0.34, 0.99)	**0.05**	0.88 (0.48, 1.61)	0.67
Other	2.08 (0.79, 5.42)	0.14	2.58 (0.90, 7.37)	0.08
**Anthropomorphic**				
BMI/(kg/m^2^)	1.07 (1.03 1.11)	**<0.001**	1.10 (1.05, 1.15)	**<0.001**
**Liver enzymes**				
GGT/(10 U/L)	1.06 (0.99, 1.13)	0.10	1.02 (0.95, 1.11)	0.55
**Serum chemistries**				
LDL/(10 mg/dL)	1.10 (1.01, 1.19)	**0.02**	1.14 (1.04, 1.24)	**0.006**
Serum glucose/(10 mg/dL)	1.06 (0.94, 1.19)	0.38	1.07 (0.94, 1.23)	0.31
Serum insulin/(10 µU/mL)	1.03 (0.98, 1.09)	0.26	1.00 (0.94, 1.07)	0.91
Uric acid/(mg/dL)	1.24 (1.04, 1.48)	**0.02**	1.34 (1.07, 1.67)	**0.01**
**Liver histology**				
Steatosis grade>33%	1.44 (0.82, 2.55)	0.21	1.82 (0.96, 3.44)	0.07
Intercept			0.003 (0.0002, 0.05)	**<0.001**
Hosmer-Lemeshow χ^2^ for model fit				0.59

Abbreviations: OR = odds ratio, CI = confidence interval, BMI = body mass index, GGT = gamma-glutamyl transpeptidase, LDL = low-density lipoprotein.

a Persistently high blood pressure was defined as systolic or diastolic blood pressure ≧95th percentile for age, sex and height or the use of antihypertensive medication at both baseline and 48 weeks. Blood pressure percentiles were computed as instructed in The Fourth Report on the Diagnosis, Evaluation, and Treatment of High Blood Pressure in Children and Adolescents.

b *P* values and 95% CI were obtained from Wald statistics.

### Clinical Hypertension

At baseline, 18% (32/177) of children with high blood pressure had a clinical diagnosis of hypertension. There were 10 children taking antihypertensive medication representing 5% of those with high blood pressure and 31% of those with a clinical diagnosis of hypertension. Over the course of one year of follow-up, there were an additional 10 children diagnosed with hypertension. Antihypertensive medications were prescribed to 2 additional children who had a clinical diagnosis of hypertension at baseline and to 2 of the children subsequently diagnosed with hypertension. At week 48, 28% (23/82) of children with persistent high blood pressure had a clinical diagnosis of hypertension and 13% were taking antihypertensive medications.

## Discussion

We studied the prevalence of high blood pressure in a longitudinal cohort study of children with NAFLD from pediatric centers across the United States. Children with NAFLD had a high rate of high blood pressure both at baseline and again at 48 weeks. The odds of having high blood pressure at baseline and high blood pressure that persisted at 48 weeks were associated with BMI, LDL-cholesterol and uric acid. Hepatic steatosis was associated with high blood pressure at baseline. Unexpectedly, girls with NAFLD had greater odds of persistent high blood pressure than boys with NAFLD.

The rates of high blood pressure in children with NAFLD exceeded what would be expected based upon the contribution of obesity alone. Population-based cohort studies estimate the prevalence of high blood pressure in obese children to be 11%. [2], [19]–[21] Although most children with NAFLD are overweight or obese, our finding that more than one of every three children with NAFLD had high blood pressure at baseline indicates that children with NAFLD are at particularly increased risk for high blood pressure. A previous single center study in overweight and obese children with biopsy-confirmed NAFLD demonstrated that mean systolic and diastolic blood pressure were significantly higher compared to overweight and obese controls without evidence of NAFLD. [9] Similarly, studies in children have shown that hepatic steatosis, independent of degree of obesity, is associated with cardiac dysfunction. [22], [23] Notably, in our cohort, children with NAFLD who had high blood pressure at baseline had higher degrees of hepatic steatosis. Data are extremely limited on the persistence of high blood pressure in children. In our study, the prevalence of 21.4% for persistent high blood pressure over 48 weeks in children with NAFLD was much higher than reported for other groups of children with longitudinal data available. For example, in the National Heart, Lung, and Blood Institute Growth and Health Study, the rate of persistent high blood pressure over 18 months in girls was 0.6% overall and 3% in obese girls. [21]

NAFLD and high blood pressure share pathophysiologic factors such as systemic oxidative stress and vascular and adipose tissue inflammation, which can produce vascular endothelial dysfunction. [24]–[28] NAFLD is associated with endothelial dysfunction independent of obesity and other metabolic syndrome features. [26] In the setting of hepatic steatosis, liver endothelial dysfunction can occur even prior to development of hepatic inflammation and fibrosis. [29] While it is not yet clear whether hypertension is a cause or consequence of endothelial dysfunction, exogenous infusion of endothelium-derived nitric oxide synthase inhibitors can produce hypertension in humans. [30] Our finding that elevated serum levels of LDL-cholesterol and uric acid were associated with increased odds for both baseline and persistent high blood pressure in this cohort also supports a possible role for underlying endothelial dysfunction. High levels of LDL cholesterol have been shown to alter the activity of endothelial-derived nitric oxide synthase. [31] Oxidized LDL is also associated with endothelial dysfunction and activation of the renin-angiotensin system. [32], [33] Likewise, elevated uric acid has been functionally linked to decreased endothelial nitric oxide synthase activity and nitric oxide production and in turn endothelial dysfunction. [34], [35] Elevated uric acid levels have been reported in children with NAFLD, possibly due to high dietary fructose intake. [36] Serum uric acid levels in childhood have been shown to predict high blood pressure beginning in childhood and persisting into adulthood. [37], [38] Thus, common pathophysiological processes may play a role in the development of both NAFLD and high blood pressure.

High blood pressure in childhood tracks into adulthood. [4], [5] High blood pressure in children with NAFLD is, therefore, likely to persist and place these children at risk for premature morbidity and mortality. Systolic blood pressure in childhood is a consistent predictor of arterial stiffness in adults. [39] Of the various metabolic syndrome factors, systolic blood pressure in childhood has the strongest correlation with coronary artery atherosclerosis in adulthood. [6] Additionally, adolescents with NAFLD have been shown to have left ventricular dysfunction compared to obese adolescents without NAFLD. [23] In adults with NAFLD, high blood pressure is linked to each of the three most common causes of death; cardiovascular disease, cancer, and liver disease. [40] Furthermore, in adults with NASH, the presence of hypertension is an important risk factor for the development of hepatocellular carcinoma. [41] Despite the potential for adverse outcomes, our data suggest that there is likely an underdiagnosis of high blood pressure in children with NAFLD. Future studies should assess interventions to improve the detection and control of high blood pressure in children with NAFLD.

The accurate assessment of blood pressure in children with NAFLD was strengthened by the use of the NASH CRN, the largest prospectively enrolled cohort of children with NAFLD with representation from across the U.S. Participants had an accurate diagnosis of NAFLD characterized in a rigorous standardized fashion. Moreover, the inclusion of longitudinal data is rare and particularly important for blood pressure research. The study was limited by the lack of three discrete measures required to confirm a clinical diagnosis of hypertension. However, large longitudinal cohort studies have shown that a single measurement of high blood pressure in childhood is strongly associated with development of hypertension in adulthood. [4] Furthermore, persistent single-time point measurements of high blood pressure in childhood carry an even greater risk of subsequent hypertension. [42] Future studies should consider the use of ambulatory blood pressure monitoring to better define the blood pressure phenotype in children with NAFLD. [43] Finally, long-term follow-up data are needed to assess for the development of adverse clinical outcomes associated with high blood pressure in children with NAFLD.

In conclusion, NAFLD is a common clinical problem that places children at substantial risk for high blood pressure, which may often go undiagnosed. Thus blood pressure evaluation and control should be an integral component of the clinical management of children with NAFLD.
